# Effect of perioperative factors on the prognosis of patients with hepatocellular carcinoma who underwent hepatectomy

**DOI:** 10.55730/1300-0144.5409

**Published:** 2022-05-12

**Authors:** Lining XU, Yingying XU, Guiping LI, Bo YANG

**Affiliations:** 1 Department of General Surgery, The Second Medical Center & National Clinical Research Center for Geriatric Diseases, Chinese PLA General Hospital, Beijing, China; 2 Department of Internal Medicine, Henan Cancer Hospital, Zhengzhou, China; 3 Department of Radiology, Hubei Province Integrated Hospital of Chinese and Western Medicine, Wuhan, China; 4 Department of Radiology, Affiliated Union Hospital, Tongji Medical College, Huazhong University of Science and Technology, Wuhan, China

**Keywords:** Hepatocellular carcinoma, prognosis, survival study, hepatectomy, perioperative

## Abstract

**Background/aim:**

Hepatic resection is a potentially curative treatment for patients with hepatocellular carcinoma (HCC). Controversy persists regarding preoperative and intraoperative characteristics related to patient survival in various medical institutes. This study aimed to evaluate the impact of preoperative and intraoperative factors on the long-term survival of patients with HCC who underwent hepatectomy.

**Materials and methods:**

Data on 455 patients with HCC who underwent hepatectomy over a 20-year period were retrospectively reviewed. Univariate and multivariate Cox regression analyses were performed for preoperative- and intraoperative-related prognostic factors.

**Results:**

The 1-, 3-, 5-, and 10-year overall survival rates of patients with HCC who underwent resection were 76.3%, 57.9%, 46.7%, and 27.4%, respectively. Multivariate analyses identified four independent predictors of long-term prognosis—sex (male versus female, hazard ratio [HR] = 2.732, p = 0.026); differentiation (poor versus well, HR = 2.037, p = 0.030); total bilirubin value (μmol/L, HR = 1.056, p = 0.033); and intraoperative blood transfusion (no transfusion versus transfusion, HR = 0.417, p = 0.002). Hepatitis virus B infection (negative versus positive, HR = 0.669, p = 0.232) and resection style (anatomical versus nonanatomical, HR = 0.698, p = 0.181) were not associated with survival.

**Conclusion:**

Based on this 20-year study, poor survival of patients with HCC who underwent hepatectomy was correlated with preoperative and intraoperative factors including male sex, poor differentiation, increased total bilirubin levels, and intraoperative blood transfusion.

## 1. Introduction

In the past, the incidence of hepatocellular carcinoma (HCC) varied widely globally, with high rates in sub-Saharan Africa, eastern and southeastern Asia, and Melanesia and low rates in Northern and Western Europe and America [1]. However, it is now rapidly becoming more prevalent in Western countries owing to the spread of hepatitis C infection and increased rate of liver cancer associated with alcohol use and nonalcoholic steatohepatitis [2]. HCC is, therefore, increasingly becoming a major contributor to the worldwide cancer burden. HCC is a histological type of primary liver cancer and originates from hepatocytes [3]. In the 17th Nationwide Follow-up Survey of Primary Liver Cancer in Japan [4], 18,213 individuals were newly registered as patients with primary liver cancer at 645 medical institutions, and 94.2% of these patients had HCC. The incidence rates of this disease have increased in many countries in recent decades [5]. As the principal histological type of liver cancer, HCC accounts for the vast majority of liver cancer diagnoses and deaths.

Currently, hepatic resection is a potentially curative treatment for patients with malignant liver lesions, especially HCC. The surgical outcome of HCC is affected by many factors [6]. However, further research is needed on ways to optimize the perioperative factors of HCC to improve the effect of surgical intervention for HCC [7].

Therefore, we retrospectively investigated the data on 455 patients with HCC who underwent hepatectomy and were followed up to identify the impact of preoperative and intraoperative factors on long-term prognosis.

## 2. Materials and methods

### 2.1. Patients

In total, 455 patients with HCC who underwent hepatectomy and subsequent follow-ups were enrolled in this investigation according to their medical records. Diagnoses were based on pathological examination. Death within 30 days of hepatic resection or at any time after 30 days was considered surgical death. Patients who experience surgical death and those who died during the hospitalization period for hepatectomy were excluded.

On admission, all selected patients had records containing information on thorough disease histories and physical examination findings. Preoperative data on patient demographic information, diagnosis, and laboratory blood analyses (bilirubin and alkaline phosphatase levels) were also collected. Intraoperative data on operation duration, estimated blood loss (EBL), resected regions, and other related items were collected from operation notes and anesthetist records. Data on blood transfusion during surgery were also recorded. Data regarding the resected specimen, including the number of tumors and size of the largest tumor, were obtained from pathology records.

Postoperative variables included complications and survival rates. Data on long-term outcomes (survival rates) were obtained through clinical follow-up and by contacting the patient and family members, if necessary.

This study was approved by the Ethics Committee of Chinese PLA General Hospital and used anonymized data.

### 2.2. Statistical analysis

All data are expressed as the percentage of patients or as mean with standard deviation (SD). Survival rate was calculated. Plots were constructed using the Kaplan–Meier method and compared using the log-rank test between groups. Cox regression was used to estimate the risk for death (hazard ratio [HR]) for prognostic factors. Differences with p < 0.05 were considered statistically significant.

## 3. Results

### 3.1. Demographic information

From January 1999 to December 2019, 455 patients with primary liver cancer underwent hepatectomy and were followed up. These patients included 398 men and 57 women, with mean (± SD) age of 50.15 ± 10.48 years (range 8–77 years). The tumor size ranged from 1 to 33 cm in diameter. Tumors measuring 5–10 cm were found in 158 (34.7%) patients, while those measuring >10 cm were found in 75 (16.5%) patients.

### 3.2. Complications

The postoperative complication rate was 9.9%; the complications are summarized in Table 1. The most common complications were related to liver cirrhosis, including pleural effusion, ascites, and sterile perihepatic fluid collection. All complications were managed successfully by general treatment, such as concentrated human albumin transfusion, adjustment of water and electrolyte balance, and/or use of diuretics.

### 3.3. Patient survival

The 1-, 3-, 5- and 10-year overall survival rates of patients with HCC who underwent resection were 76.3%, 57.9%, 46.7%, and 27.4%, respectively. Several preoperative and operative variables associated with survival rates in univariate analysis are summarized in Table 2, including sex (p = 0.001), alpha-fetoprotein (p < 0.001), total bilirubin (p < 0.001), alkaline phosphatase (p = 0.013), Child–Pugh score (p = 0.003), tumor size (p < 0.001), number of lesions (p = 0.006), differentiation (p < 0.001), adjacent organ resection (p < 0.001), ≥ 3 segments resected (p = 0.048), resection style (p = 0.002), operative duration (p = 0.002), intraoperative blood loss (p < 0.001), and intraoperative blood transfusion (p = 0.001). Multivariate analyses (as shown in Tables 3 and 4) identified four independent predictors of long-term prognosis—sex (male versus female, HR = 2.732, p = 0.026), differentiation (poor versus well, HR = 2.037, p = 0.030), intraoperative blood transfusion (no transfusion versus transfusion: HR = 0.417, p = 0.002), and total bilirubin level (μmol/L: HR = 1.056, p = 0.033). Hepatitis B virus (HBV) infection (negative versus positive: HR = 0.669, p = 0.232) and resection style (anatomical versus nonanatomical: HR = 0.698, p = 0.181) were not correlated with survival.

## 4. Discussion

### 4.1. Complications of hepatectomy among patients with HCC

Hepatectomy is a complex and difficult surgical technique. Despite technical advances and extensive experience with liver resection in specialized centers, hepatectomy is associated with relatively high rates of postoperative morbidity. The four potentially devastating complications of liver resection include postoperative hemorrhage, venous thromboembolism, bile leakage, and posthepatectomy liver failure [8]. The risk factors and management of these complications were herein explored, stressing the importance of identifying preoperative factors that could decrease the risk for these potentially fatal complications. Some perioperative factors responsible for the increase in complications are as follows [9]: American Society of Anesthesiologists classification; patient age, platelet count, intraoperative EBL, and tumor number. In this study, the postoperative complication rate for HCC was 9.9%. The most common complications were related to liver cirrhosis, including pleural effusion, ascites, and sterile perihepatic fluid collection. All complications were managed successfully by general treatment, such as concentrated human albumin transfusion, adjustment of water and electrolyte balance, or use of diuretics.

### 4.2. Overall survival rates of patients with HCC who underwent hepatectomy

The survival rates for HCC after liver resection vary among medical institutes. Chen [10] reported that the mean expected survival times and survival rates at 5 years were 77.8 months and 47.1%, respectively. In a study by Lee [11], the 1-, 3-, and 5-year overall survival rates were 91.9%, 78.9%, and 69.5%, respectively, and the 1-, 3-, and 5-year recurrence-free survival rates were 71.7%, 51.7%, and 43.7%, respectively. A study involving 1330 consecutive patients reported that the overall survival rates at 1, 3, and 5 years were 91.2%, 63.3%, and 36.9%, respectively, and the disease-free survival rates at 1, 3, and 5 years were 67.7%, 33.7%, and 13.8%, respectively [12].

### 4.3. Controversial clinical characteristics associated with patient survival

The incidence of HCC coincident with liver cirrhosis is increasing in some high-epidemic areas of HBV infection. In this study, hepatitis B surface antigen was tested in 423 patients, among whom 337 (79.7%) showed positivity. The 1-, 3-, 5- and 10-year survival rates in the HBV group were 75.1%, 56.0%, 45.4%, and 28.4%, respectively, and those in the non-HBV group were 82.6%, 64.8%, 50.7%, and 30.6%, respectively. There were no significant differences between their survival rates (p = 0.073). However, a study by Wu [13] indicated that the 1- and 3-year recurrence-free survival rates of HBV-negative patients were significantly better than those of HBV-positive patients (66% and 25% versus 89% and 70%). However, there were no significant differences in the 1-, 3-, and 5-year overall survival rates between the groups.

The liver resection style was an important factor associated with prognosis. It is unclear whether hepatectomy for HCC should be performed as an anatomical resection (AR) or a non-AR (NAR). No randomized controlled trials addressing this topic are currently available. Jiao [14] searched for articles investigating AR versus NAR for HCC published between January 1998 and December 2018 in the PubMed, Cochrane Library, EMBASE, and Wanfang databases. Metaanalysis was performed on patient characteristics, tumor characteristics, operative characteristics, perioperative outcomes, and long-term outcomes. Thirty-eight studies involving 9122 patients were included, among whom 5062 were included in the AR group and 4060 were included in the NAR group. The AR group exhibited better 1-, 3-, and 5-year overall survival and disease-free survival rates than the NAR group.

Our results were different from these findings. In our study, the 1-, 3-, 5- and 10-year survival rates were 79.5%, 61.9%, 49.7%, and 29.7%, respectively, in the NAR group and 65.4%, 44.4%, 36.5%, and 21.7%, respectively, in the AR group. Univariate analysis indicated that survival rates in the NAR group were significantly higher than those in the AR group (p = 0.002). However, multivariate analysis indicated that survival was not correlated with resection style (p = 0.181). The advantages of NAR appear to be twofold—conserving liver function and reducing the dangers associated with more extensive liver resections. This is particularly advantageous in patients who may be at higher risk for AR, such as those with cirrhosis and those with a small remnant liver. Some studies have indicated that small-for-size livers are more conducive to tumor growth and metastasis [15, 16]. Liver regeneration after major surgery may activate occult micrometastases and facilitate tumor growth, leading to liver tumor recurrence [17].

The incidence rate of not only liver cirrhosis but also HBV infection is high in China. Liver cirrhosis is the primary cause of liver cancer in China. Liver function reserve in patients with cirrhosis is poor. The use of AR will result in more liver tissue loss than the use of NAR, and the probability of postoperative liver insufficiency will increase. Therefore, consistent with our study results, AR has no advantage over NAR in Chinese patients with liver cirrhosis. However, given that hepatectomy is mostly performed in the absence of cirrhosis in Europe, the United States, and other countries, AR has been recommended in literature reports involving patients outside of China as research subjects.

In a retrospective study, Wong [18] reported that elevated alpha-fetoprotein levels, low albumin levels, and tumors measuring >5 cm were associated with increased 1-year mortality after hepatic resection for early-stage HCC. Kondo [19] reported that alkaline phosphatase level (> 125 U/L), alpha-fetoprotein level (within 20–400 or >400 ng/mL), protein induced by vitamin K absence-II (within 40–400 or > 400 mAU/mL), tumor number, diameter, pseudocapsule, tumor growth pattern, and intratumor hemorrhage were independent prognostic factors for HCC. In our study, multivariate analyses indicated that the total bilirubin value (μmol/L, HR = 1.056, p = 0.033) was an independent predictor of poor survival for HCC, and Child–Pugh status was not correlated with survival.

Limited studies have reported correlations between long-term survival and intraoperative factors, such as blood loss and blood transfusion. Several studies have suggested that preoperative blood loss or transfusions have a negative impact on postoperative outcomes. However, it is unclear whether this is due to a real cause–effect relationship or merely the result of a more complicated surgery. The effect of blood transfusions on tumor recurrence and long-term mortality is much less clear, and evidence varies depending on the type of malignancy [20]. In our study, univariate analysis indicated that the survival rates among cases involving intraoperative blood loss <400 mL were significantly higher than those among cases involving intraoperative blood loss was >400 mL (p < 0.001). The survival rates in the no intraoperative blood transfusion group were significantly higher than those in the intraoperative blood transfusion group (p = 0.001). However, multivariate analysis indicated that only intraoperative blood transfusion was a prognostic indicator associated with a higher risk for death (no transfusion versus transfusion, HR = 0.417, p = 0.002).

### 4.4. Conclusion

The prognostic factors for HCC after hepatectomy are controversial. In our 20-year study, the poor survival of patients with HCC who underwent hepatectomy was correlated with preoperative and intraoperative factors including male sex, poor differentiation, increased total bilirubin levels, and intraoperative blood transfusion, but not with HBV infection or resection style.

## Figures and Tables

**Table 1:** Postoperative complications.

Complication	Number	Ratio (%)
Abdomen		
Incisional wound infection	1	0.22
Pelvic cavity fluid collection	1	0.22
Retroperitoneum fluid collection	1	0.22
Stress ulcer	1	0.22
Alimentary tract hemorrhage	1	0.22
Abdominal cavity/raw surface bleeding	1	0.22
Hepatic inadequacy	1	0.22
Bile leakage	2	0.44
Wound liquefaction	2	0.44
Renal inadequacy	3	0.66
Incision disruption	4	0.88
Periliver fluid collection (sterile)	8	1.76
Ascites	12	2.64
Pulmonary and cardiovascular		
Deep venous thrombosis (lower extremity)	2	0.44
Pneumothorax	2	0.44
Atelectasis	2	0.44
Pneumonia	6	1.32
Pleural effusion	19	4.18
Others		
Fever of unknown origin	2	0.44

**Table 2:** Univariate analysis of factors associated with survival rate

Variable	Number	Survival rate (%)				p-value*
		1-year	3-year	5-year	10-year	
Sex						
Male	398	74.1	55.2	44.0	24.8	0.001
Female	57	91.2	77.0	65.3	46.5	
Age (years)						
≤40	67	70.1	56.4	50.0	32.3	0.309
41–50	177	78.5	55.3	45.1	30.8	
51–60	127	74.8	55.0	42.1	22.3	
>60	84	88.1	69.0	55.4	31.2	
Alpha-fetoprotein (ng/ml)#						
≤20	98	84.7	74.2	61.0	26.8	0.000
>20	217	74.7	49.7	39.0	20.1	
Total bilirubin (μmol/L)#						
≤21	258	80.2	63.2	54.1	35.4	0.000
>21	144	70.1	46.1	32.9	0	
Alkaline phosphatase (U/L)#						
≤130	292	77.1	58.6	49.4	28.9	0.013
>130	56	67.9	46.2	34.6	19.2	
Hepatitis B surface antigen#						
Negative	86	82.6	64.8	50.7	30.6	0.073
Positive	337	75.1	56.0	45.4	28.4	
Child–Pugh score#						
5	293	79.5	60.5	51.6	33.8	0.003
6	74	68.9	50.7	41.5		
7	25	60	51.7	35.2		
8	12	34.3				
9	4	50				
Largest tumor size (cm)						
≤5	222	85.6	70.0	57.0	30.1	0.000
5–10	158	70.9	47.5	39.7	24.7	
>10	75	60.0	43.8	33.0	24.4	
Number of lesions#						
Single	400	77.5	59.9	49.2	28.8	0.006
Multiple	54	66.7	43.8	24.7	18.5	
Differentiation#						
Well	69	91.3	69.2	64.1	46.7	0.000
Moderate	183	78.7	58.5	45.3	14.0	
Poor	86	58.1	40.2	37.3	22.1	
Previous abdominal surgery						
No	409	76.8	57.1	46.8	26.2	0.465
Yes	46	71.7	60.4	46.3	46.3	
Laparoscopic resection						
No	437	75.7	57.1	45.9	27.0	0.140
Yes	18	83.3	72.2	66.7		
Adjacent organ resection						
No	450	76.9	58.5	47.2	27.7	0.000
Yes	5	20.0				
≥ 3 segments resected						
No	303	79.9	61.1	50.3	29.9	0.048
Yes	152	69.1	51.7	38.3	21.7	
Resection style						
Nonanatomical	351	79.5	61.9	49.7	29.7	0.002
Anatomical	104	65.4	44.4	36.5	21.7	
Operative duration (min)						
≤ 180	267	80.9	62.2	52.6	33.3	0.002
> 180	188	69.7	51.8	38.4	19.7	
Intraoperative blood loss (mL)						
≤ 400	328	81.7	61.9	51.1	30.5	0.000
> 400	127	62.2	47.3	34.9	18.4	
Intraoperative blood transfusion						
No	262	83.6	63.0	52.3	39.6	0.001
Yes	193	66.3	51.0	38.9	20.4	
Incisional margin						
Residual tumor	4	50.0	50.0	27.2	0	0.943
No residual tumor	451	76.3	57.7	46.7		
Complication						
No	410	77.3	57.9	47.0	28.0	0.637
Yes	45	66.7	57.6	43.7	24.3	

* Log-rank (Mantel–Cox) test

# Missing data

**Table 3:** Multivariate analysis of factors associated with survival (numeration data).

Variable	Hazard ratio (95% confidence interval)	*P* value*
Sex		
Female	1.000-	
Male	2.732 (1.129–6.613)	0.026
HBsAg		
Positive	1.000-	
Negative	0.669 (0.347–1.292)	0.232
Differentiation		
Well	1.000-	
Moderate	1.237 (0.702–2.178)	0.462
Poor	2.037 (1.071–3.877)	0.030
Previous abdominal surgery		
Yes	1.000-	
No	1.716 (0.706–4.170)	0.233
≥3 segments resected		
Yes	1.000-	
No	1.603 (0.988–2.601)	0.056
Resection style		
Anatomical	1.000-	
Nonanatomical	0.698 (0.412–1.182)	0.181
Intraoperative blood loss (ml)		
≤400	1.000-	
>400	1.313 (0.717–2.405)	0.378
Intraoperative blood transfusion		
Yes	1.000-	
No	0.417 (0.239–0.726)	0.002
Complication(s)		
Yes	1.000-	
No	0.769 (0.368–1.603)	0.483

* Cox regression

HBsAg, hepatitis B surface antigen

**Table 4:** Multivariate analysis of factors associated with survival (measurement data).

Variable	Hazard ratio (95% CI)	p-value*
Age (years)	1.008 (0.986–1.030)	0.481
Alpha-fetoprotein (ng/mL)	1.000 (1.000–1.000)	0.481
Total bilirubin (μmol/L)	1.056 (1.004–1.110)	0.033
Alkaline phosphatase (U/L)	1.003 (0.999–1.007)	0.143
Child–Pugh score	0.955 (0.679–1.343)	0.791
Largest tumor size (cm)	1.003 (0.997–1.009)	0.366
Operative time (min)	1.000 (0.998–1.003)	0.766

* Cox regression

## References

[1] Amini M Looha MA Zarean E Pourhoseingholi MA Global pattern of trends in incidence, mortality, and mortality-to-incidence ratio rates related to liver cancer, 1990–2019: a longitudinal analysis based on the global burden of disease study BMC Public Health 2022 22 1 604 10.1186/s12889-022-12867-w 35351047 PMC8961994

[2] Asrani SK Devarbhavi H Eaton J Kamath PS Burden of liver diseases in the world Journal of Hepatology 2019 70 1 151 171 10.1016/j.jhep.2018.09.014 30266282

[3] Augustine MM Fong Y Epidemiology and risk factors of biliary tract and primary liver tumors Surgical Oncology Clinics of North America 2014 23 2 171 188 10.1016/j.soc.2013.10.001 24560105

[4] Ikai I Arii S Okazaki M Okita K Omata M Report of the 17th Nationwide Follow-up Survey of Primary Liver Cancer in Japan Hepatology Research 2007 37 9 676 691 10.1111/j.1872-034X.2007.00119.x 17617112

[5] McGlynn KA Petrick JL El-Serag HB Epidemiology of Hepatocellular Carcinoma Hepatology 2021 73 Suppl 1 4 13 10.1002/hep.31288 PMC7577946 32319693

[6] Xu LN Xu YY Gao DW Impact of operative and peri-operative factors on the long-term prognosis of primary liver cancer patients undergoing hepatectomy Journal of Huazhong University of Science and Technology (Medical Sciences) 2016 36 4 523 528 10.1007/s11596-016-1619-2 27465327

[7] Walcott-Sapp S Billingsley KG Preoperative optimization for major hepatic resection Langenbecks Archives of Surgery 2018 403 1 23 35 10.1007/s00423-017-1638-x 29150719

[8] Russell MC Complications following hepatectomy Surgical Oncology Clinics of North America 2015 24 1 73 96 10.1016/j.soc.2014.09.008 25444470

[9] Hoffmann K Hinz U Stravodimos C Knoblich T Schön MR Risk assessment for liver resection Surgery 2018 164 5 998 1005 10.1016/j.surg.2018.06.024 30107885

[10] Chen S Jin H Dai Z Wei M Xiao H Liver resection versus transarterial chemoembolization for the treatment of intermediate-stage hepatocellular carcinoma Cancer Medicine 2019 8 4 1530 1539 10.1002/cam4.2038 30864247 PMC6488138

[11] Lee EC Kim SH Park H Lee SD Lee SA Survival analysis after liver resection for hepatocellular carcinoma: A consecutive cohort of 1002 patients Journal of Gastroenterology and Hepatology 2017 32 5 1055 1063 10.1111/jgh.13632 27797420

[12] Li J Huang L Yan J Qiu M Yan Y Liver resection for hepatocellular carcinoma: personal experiences in a series of 1330 consecutive cases in China ANZ Journal of Surgery 2018 88 10 713 717 10.1111/ans.14381 29363237

[13] Wu ZF Xu Z Li WS Zhang HB Yang N Impact of occult hepatitis B virus infection on outcome after resection for non-B non-C hepatocellular carcinoma Journal of Surgery Research 2015 193 1 153 160 10.1016/j.jss.2014.07.021 25128925

[14] Jiao S Li G Zhang D Xu Y Liu J Anatomic versus non-anatomic resection for hepatocellular carcinoma, do we have an answer? A meta-analysis International Journal of Surgery 2020 80 1 243 255 10.1016/j.ijsu.2020.05.008 32413500

[15] Shoreem H Gad EH Soliman H Hegazy O Saleh S Small for size syndrome difficult dilemma: Lessons from 10 years single centre experience in living donor liver transplantation World Journal of Hepatology 2017 9 21 930 944 10.4254/wjh.v9.i21.930 28824744 PMC5545138

[16] Kornberg A Witt U Kornberg J Friess H Thrum K Extended ischemia times promote risk of HCC recurrence in liver transplant patients Digestive Diseases and Sciences 2015 60 9 2832 2839 10.1007/s10620-015-3541-z 25630421

[17] Li HM Ye ZH Microenvironment of liver regeneration in liver cancer Chinese Journal of Integrative Medicine 2017 23 7 555 560 10.1007/s11655-017-2806-0 28523536

[18] Wong LL Hernandez BY Shvetsov YB Kawano Y Tang ZY Liver resection for early hepatocellular cancer: Comparison of centers in 3 different countries World Journal of Hepatology 2016 8 31 1327 1335 10.4254/wjh.v8.i31.1327 27872684 PMC5099585

[19] Zhou Q Zhou C Yin Y Chen W Liu C Development and validation of a nomogram combining hematological and imaging features for preoperative prediction of microvascular invasion in hepatocellular carcinoma patients Annals of Translational Medicine 2021 9 5 402 10.21037/atm-20-4695 33842623 PMC8033313

[20] Bennett S Baker L Shorr R Martel G Fergusson D The impact of perioperative red blood cell transfusions in patients undergoing liver resection: a systematic review protocol Systematic Reviews 2016 5 38 10.1186/s13643-016-0217-5 26926289 PMC4770706

